# Exploration of chemical space with partial labeled noisy student self-training and self-supervised graph embedding

**DOI:** 10.1186/s12859-022-04681-3

**Published:** 2022-05-02

**Authors:** Yang Liu, Hansaim Lim, Lei Xie

**Affiliations:** 1 Department of Computer Science, Hunter College, The City University of New York, 695 Park Ave, New York, NY 10065 USA; 2 The Graduate Center, The City University of New York, 356 5th Ave, New York, NY 10016 USA

**Keywords:** Drug-target interaction, Drug discovery, Drug toxicity, Drug metabolism, Deep neural network, Graph neural network, Semi-supervised learning, Self-supervised learning, Chemical embedding, Artificial intelligence

## Abstract

**Background:**

Drug discovery is time-consuming and costly. Machine learning, especially deep learning, shows great potential in quantitative structure–activity relationship (QSAR) modeling to accelerate drug discovery process and reduce its cost. A big challenge in developing robust and generalizable deep learning models for QSAR is the lack of a large amount of data with high-quality and balanced labels. To address this challenge, we developed a self-training method, Partially LAbeled Noisy Student (PLANS), and a novel self-supervised graph embedding, Graph-Isomorphism-Network Fingerprint (GINFP), for chemical compounds representations with substructure information using unlabeled data. The representations can be used for predicting chemical properties such as binding affinity, toxicity, and others. PLANS-GINFP allows us to exploit millions of unlabeled chemical compounds as well as labeled and partially labeled pharmacological data to improve the generalizability of neural network models.

**Results:**

We evaluated the performance of PLANS-GINFP for predicting Cytochrome P450 (CYP450) binding activity in a CYP450 dataset and chemical toxicity in the Tox21 dataset. The extensive benchmark studies demonstrated that PLANS-GINFP could significantly improve the performance in both cases by a large margin. Both PLANS-based self-training and GINFP-based self-supervised learning contribute to the performance improvement.

**Conclusion:**

To better exploit chemical structures as an input for machine learning algorithms, we proposed a self-supervised graph neural network-based embedding method that can encode substructure information. Furthermore, we developed a model agnostic self-training method, PLANS, that can be applied to any deep learning architectures to improve prediction accuracies. PLANS provided a way to better utilize partially labeled and unlabeled data. Comprehensive benchmark studies demonstrated their potentials in predicting drug metabolism and toxicity profiles using sparse, noisy, and imbalanced data. PLANS-GINFP could serve as a general solution to improve the predictive modeling for QSAR modeling.

## Background

Recent advances in deep learning have shown promises in quantitative structure–activity relationship (QSAR) modeling for accelerating drug discovery [1]. However, several challenges remain in the successful application of deep learning to QSAR. A general problem for almost all supervised deep learning is that the amount of high-quality labeled data is usually limited. To train an accurate, robust, and generalizable model using deep learning, it is not surprising to use millions of labeled training data. Even the most advanced high throughput experimental methods today cannot meet the huge data demand. Thus, methods are needed to sufficiently exploit the labeled pharmacological data and unlabeled chemical space to improve the performance of deep learning models. Autoencoder [2, 3] and variant autoencoder [4, 5] are widely used models, which can be pre-trained with unlabeled data in order to better represent input data features by mapping the sparse sample space to a continuous latent space. Multi-task models try to find a better latent space by combining datasets with labels for different tasks [6, 7]. Recently, self-supervised learning, which has achieved tremendous success in Natural Language Processing (NLP) [8], is extended to graph-structured data [9]. The self-training method is another method to explore unlabeled data space. It uses the “teacher” model trained with a small amount of data to label the unlabeled data and recursively train “student” models that perform better than the “teacher” model [10]. Recently, Google developed a “Noisy Student” self-training method, in which they used a simple teacher model to train a series of student models with gradually increasing complexity [11]. In their experiments, the Noisy Student-trained models showed not only better performance but also more robust than standard convolutional neural network (CNN) model. Despite their successes in imaging processing and NLP, few works have applied the concept of self-training to QSAR modeling given that billions of chemical compounds are not associated with any labels. In addition to the huge amount of unlabeled data, bioassay data is often highly imbalanced. Conventional over-sampling or over-sampling techniques such as SMOTE [12], which rely on the similarity between samples, maybe not sufficient in addressing this problem. On one hand, a small modification in a chemical structure could result in a dramatic change in bioactivity [13]. On the other hand, highly dissimilar chemical compounds could have similar bioactivity [14]. Therefore, new techniques are needed to address sparse, biased, and imbalanced sample problem.

In this work, for the first time, we design a combined self-training and self-supervised learning strategy to explore unlabeled chemical space for improving predictive modeling in QSAR. We develop a new self-training method: Partially LAbeled Noisy Student (PLANS). Furthermore, we propose a novel self-supervised graph embedding approach GINFP for chemical representations using unlabeled data. The GINFPs were constructed by learning chemical molecule graphs with GIN [15], a Graph Neural Network model. GIN represents the state-of-the-art GNN model in GNN [9, 16]. In Hu’s work, it shows that GIN outperforms other popular GNNs like GCN, GraphSAGE, and GAT in the chemical structure representation [9]. Furthermore, GNN could be more powerful than chemical fingerprints such as ECFP [17]. To reconstruct a molecule without labels, we can use a graph autoencoder. However, a conventional graph autoencoder cannot represent substructure information that is critical for molecular properties. Our proposed GINFP constructs corresponding ECFPs that encode substructure information for a molecule. The hidden embeddings of the graphs were used as input for classification tasks. A similar method was leveraged for GNN model pretraining and demonstrated that it is more effective than the graph autoencoder in improving prediction performance [18]. We also use the PLANS-GINFP to address the problem of sample imbalance.

To demonstrate the value of PLANS-GINFP, we applied our method to the prediction of Cytochrome P450 (CYP450) chemical binding profile and chemical toxicities. CYP450 is a protein enzyme family that catalyzes hydroxyl group incorporation with heme as a cofactor. In humans, CYP450s, as terminal oxidase in the electron transferring chain, participate in a wide range of metabolism processes including hormone synthesis, fatty acids synthesis, steroids oxidization, etc. [19]. CYP450s play key roles in drug metabolism. The activation or deactivation of approximately 75% of drugs is mediated by CYP450s [20]. The human genome has 57 CYP450 genes expressing proteins that share similar folding [21]. Therefore, CYP450s are one of the major reasons for adverse drug interactions. Furthermore, the polymorphism of CYP450s accounts for the individual difference in drug responses. Thus, studies in the binding profile of drugs to CYP450s are also a fruitful source for pharmacogenomics. The binding profile of a large number of chemical molecules with CYP450 remains unknown. However, few machine learning algorithms can reliably predict CYP450-drug interactions. Five CYP450 isoforms, 1A2, 2C9, 2C19, 2D6, and 3A4 are chosen to be the targets for chemical compounds in this work, which play the most important roles in drug metabolism [22]. Comprehensive benchmark studies demonstrated that the PLANS-GINFP significantly improved the performance of the CYP450 binding profile prediction.

Drug toxicity is a challenge for new drug development. It is time and labor costly to test the toxicity of the drug candidates in clinical trials and could be potentially harmful to the patients who participated in the trials. With PLANS, we predicted toxicities of drugs in the Tox21 dataset, which contains the information of 7,831 chemical molecules against 12 toxicity-related pathways [23]. In our experiments, we observed the performance improvement with PLANS comparing to MLP baselines is dramatic. It demonstrated the power of the PLANS model on imbalanced datasets such as Tox21. Our experiment results demonstrate the potential of our model in facilitating drug design by predicting CYP450 binding profiles and toxicity of chemical compounds. Furthermore, as an agnostic model, PLANS could be applied to other deep learning tasks for drug design using any neural network architectures and with a limited amount of noisy data.

## Results

To assess the feasibility of the Noisy Student method in QSAR modeling for drug design, we created a CYP450s benchmark dataset, which includes CYP1A2, CYP2C9, CYP2C19, CYP2D6, and CYP3A4 as targets based on the experiment published in [24]. First, we tested the prediction performance of four conventional ML algorithms and compared them to the Noisy Student trained multilayer perceptron (MLP) model. Then we introduced PLANS to improve the performance. At last, to further boost the performance, we developed two strategies: (1) balance the training set with PLANS, and (2) use a continuous fingerprint generated by a pre-trained GNN model.

### Self-training with noisy students improves the performance of QSAR modeling

Besides neural network models, we tested three types of most widely used machine learning methods, Support Vector Machine (SVM), Random Forest (RF), and gradient boosting. For gradient boosting, we chose AdaBoost and XGBoost. The configurations of the models can be found in the baseline models in “Methods” section The top rows of Table 1 shows the classification results using above four models.

**Table 1:** Classification results of the baseline models, MLP with NS, and MLP with PLANS using ECFP for the representation of chemical structures

		Accuracy	Precision	Recall	F1
Cyp450	SVM	56.37 ± 0.93	0.53 ± 0.01	0.81 ± 0.01	0.64 ± 0.01
	RF	54.44 ± 0.98	0.42 ± 0.01	0.81 ± 0.02	0.55 ± 0.01
	AdaBoost	52.40 ± 1.01	0.25 ± 0.02	**0.89 ± 0.01**	0.39 ± 0.02
	XGBoost	55.13 ± 1.21	0.57 ± 0.01	0.75 ± 0.01	0.65 ± 0.01
	MLP	51.97 ± 1.12	0.64 ± 0.03	0.72 ± 0.02	0.68 ± 0.02
	MLP + mixup	54.28 ± 0.79	0.60 ± 0.02	0.73 ± 0.01	0.66 ± 0.01
	MLP + NS	56.11 ± 1.63	0.64 ± 0.01	0.76 ± 0.02	0.69 ± 0.01
	MLP + mixup + NS	56.48 ± 1.45	0.60 ± 0.04	0.76 ± 0.02	0.67 ± 0.02
	MLP + PLANS	58.94 ± 0.96	0.72 ± 0.02	0.78 ± 0.01	**0.75 ± 0.01**
	MLP + PLANS + mixup	58.04 ± 0.70	0.69 ± 0.02	0.76 ± 0.01	0.72 ± 0.01
	MLP + PLANS + balancing	59.02 ± 1.12	**0.73 ± 0.03**	0.76 ± 0.02	**0.75 ± 0.01**
	MLP + PLANS + balancing + mixup	**59.25 ± 1.04**	0.68 ± 0.03	0.78 ± 0.01	0.73 ± 0.01

		AP	F1
Tox21	MLP	0.03 ± 0.01	–
	MLP + mixup	0.12 ± 0.01	0.02 ± 0.01
	MLP + NS	0.03 ± 0.004	–
	MLP + mixup + NS	0.13 ± 0.01	0.08 ± 0.03
	MLP + PLANS	0.14 ± 0.01	0.04 ± 0.01
	MLP + PLANS + mixup	**0.20 ± 0.03**	**0.25 ± 0.02**
	MLP + PLANS + balancing	0.16 ± 0.02	0.09 ± 0.04
	MLP + PLANS + balancing + mixup	0.20 ± 0.02	0.20 ± 0.02

The best performance is highlight in bold. The upper part shows the results for the CYP450 datasets and the lower part shows the results for Tox21 dataset. Note that underlined AdaBoost achieves the best recall performance. However, it is heavily affected by data imbalance. The recision and F1 scores of AdaBoost were much lower than other models

SVM achieved the best accuracy while XGBoost got the best precision and F1 scores. It was worth noting that while the AdaBoost model got the highest recall value, its precision and F1 scores were much lower than the other three models. It suggested that the AdaBoost model was affected the most by the data imbalance, which was a known disadvantage of AdaBoost. XGBoost, on the other hand, got well-balanced precision and recall. Thus, its F1 score was the highest among the baseline models.

To evaluate the feasibility of the Noisy Student (NS) method, we trained an MLP model with and without NS. When using NS, the Small, Medium, and Large models, as described in “Self-training with noisy student” section in “Methods” were trained iteratively with the fully labeled data and evaluation was done with the Large model. When not using NS, the Large model was trained and evaluated directly with the same training and testing sets as the model trained with NS. As shown in the second part of the CYP450 section in Table 1, all of the MLP models showed more balanced behaviors. Even the MLP without NS achieved higher precision and F1 scores than the XGBoost model. After introducing the mixup method, the MLP model without NS was already able to get an almost equivalent accuracy to XGBoost while keeping a balanced performance on precision and recall. After introducing NS, the MLP models surpassed the XGBoost model with a margin of 1.35% on accuracy and up to 4.0% on the F1 score.

To further evaluate the contribution of NS, we applied the model to another widely used baseline dataset, Tox21. Since Tox21 was a 12-class multi-label classification task, and the labels were highly imbalanced, we used average precision (AP) instead of precision and recall to evaluate the prediction. For the same reason, we only tested MLP models on the dataset. The results showed that NS itself failed to improve the prediction performance. Instead, the mixup played an important role in Tox21 predictions. Without the mixup, the model failed to converge and gave no positive predictions. With the mixup, the model successfully recalled some positive samples, while the AP score increased by a large margin (Table 1, Tox21 section). The mixup was critical for the Tox21 dataset because the labels were highly imbalanced that only approximately one-tenth of the samples were positive. Thus, data augmentation with the mixup made the model predict positive more likely and give higher F1 and AP scores.

### PLANS exploits partial labels and improves the performance of self-training with NS

Our CYP450s dataset and the Tox21 baseline dataset contain many partially labeled samples. This is also the case for many pharmacological datasets [25]. Several methods have been developed to exploit partially labeled data [26, 27]. Generally speaking, these studies were trying to maximize the margin between candidate labels and the negative labels. NS provides a more efficient way to dig out the information in partially labeled samples. Especially when the dataset contains both fully labeled samples and partially labeled samples as our CYP450s and Tox21 datasets. As described in the methods section, we developed PLANS that utilized the teacher model trained with fully labeled data to generate semi-solid labels for the partially labeled data. Then the partially labeled data were combined with the fully labeled data to train the student model. The results were shown in the third part of the CYP450 section and the second part of the Tox21 section in Table 1.

Compared with the original NS-trained model, Table 1 shows that the prediction accuracy for CYP450s with the PLANS-trained model improved about 5.0% (*p* = 0.005) and 2.8% (*p* = 0.031) without the mixup and with the mixup, respectively. Precision, recall, and F1 score also significantly increased. Specifically, the precision increased 15.0% (without the mixup) and 12.5% (with the mixup), respectively. The performance improvement in Tox21 was more significant. The AP increased from 0.03 to 0.14 without the mixup and from 0.13 to 0.20 with the mixup, respectively.

Surprisingly, the mixup augmentation approach made the model perform worse for CYP450s. A possible explanation is both partial labeling and mixup introduce noises into the training set. When a partially labeled sample was added to another sample, the noise is so large that it confuses the model. In contrast, the mixup augmentation significantly improved the performance of the Tox21 dataset. The AP score improved from 0.14 to 0.20 with the partially labeled data. The reduced imbalance in the dataset could offset the noisiness introduced by the mixup augmentation.

### Improve performance with the GINFP

The PLANS method already showed a strong ability to improve the performance of the neural network model. However, a downside of the previous experiments is the input is a highly sparse fingerprint vector with high dimensions, which could result in a large and hard to train neural network model. Thus, to test if a denser and lower dimension fingerprint can further improve the performance, we used a pre-trained Graph Isomorphism Network (GIN) to generate 300-dimension fingerprints with continuous values. We named these fingerprints as GINFP. The details about how to train the GIN model and how to generate GINFPs can be found in the methods GIN fingerprints section.

**Fig. 1:**
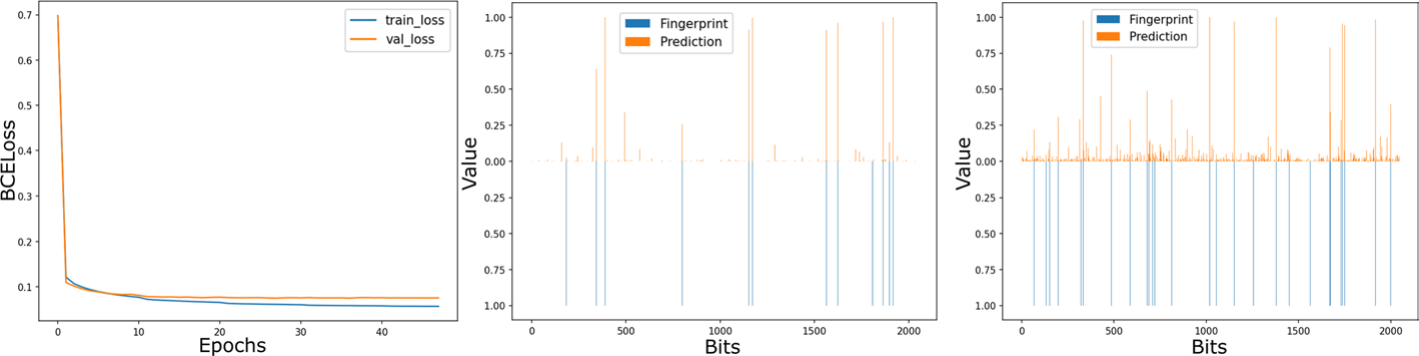
GINFP training loss and ECFP constructions. Every bit of ECFP and the predicted ECFP constructed from GINFP are shown as bars below and above the x-axis, respectively. The values of predicted ECFP are after sigmoid activation

We first evaluate if GIN can reconstruct ECFP. As shown in Fig. 1, the weights of the GIN model that we used to reconstruct ECFP converged at epoch 22. Training and evaluation loss stay stable in the whole training process. After pretraining the model, we tested it with several testing cases. The results show our model was able to precisely reconstruct both sparse ECFP (Fig. 1, middle panel) and relatively denser ECFP (Fig. 1, right panel).

We conducted the same experiments as above with all the conventional models and MLPs to evaluate if GINFP can improve the performance. One important difference is that the MLP has fewer parameters since the input has a lower dimension. As expected, GINFPs improved the performance of most of the models with a margin of ~ 2% for CYP450s (Table 2). The improvement by GINFP is more significant for the Tox21 dataset. The AP increased from 0.03 to 0.08 and from 0.14 to 0.23 for NS and PLANS, respectively. This result confirmed that the continuous fingerprint GINFPs that derived directly from molecule graphs were indeed more informative for computational models. The only model that showed a performance drop is AdaBoost. In the case of GINFP, the mixup resulted in a mild performance drop for CPY450s. This further confirmed that mixup may introduce many noises in some cases. A better data augmentation method for orientation invariant vectors may further improve the performance of PLANS.

**Table 2:** Classification results of the baseline models, MLP with NS, and MLP with PLANS using GINFP for the representation of chemical structures

		Accuracy	Precision	Recall	F1
Cyp450	SVM	58.19 ± 0.81	0.68 ± 0.01	0.77 ± 0.01	0.72 ± 0.01
	RF	54.38 ± 0.78	0.46 ± 0.01	0.79 ± 0.02	0.58 ± 0.01
	AdaBoost	48.13 ± 3.86	0.23 ± 0.09	0.83 ± 0.14	0.35 ± 0.07
	XGBoost	54.93 ± 0.78	0.59 ± 0.02	0.76 ± 0.01	0.66 ± 0.01
	MLP	57.31 ± 1.47	0.74 ± 0.02	0.75 ± 0.02	0.74 ± 0.01
	MLP + mixup	57.42 ± 0.46	0.65 ± 0.03	0.78 ± 0.02	0.71 ± 0.01
	MLP + NS	59.83 ± 0.41	0.70 ± 0.02	0.79 ± 0.01	0.74 ± 0.01
	MLP + mixup + NS	58.50 ± 0.48	0.65 ± 0.00	0.78 ± 0.01	0.71 ± 0.01
	MLP + PLANS	60.61 ± 1.00	**0.77 ± 0.01**	0.79 ± 0.01	**0.78 ± 0.01**
	MLP + PLANS + mixup	59.95 ± 1.41	0.72 ± 0.02	0.79 ± 0.01	0.75 ± 0.01
	MLP + PLANS + balancing	**61.58 ± 0.87**	0.75 ± 0.02	**0.80 ± 0.02**	**0.78 ± 0.01**
	MLP + PLANS + balancing + mixup	60.58 ± 1.38	0.73 ± 0.02	0.78 ± 0.02	0.76 ± 0.01

		AP	F1
Tox21	MLP	0.04 ± 0.01	–
	MLP + mixup	0.15 ± 0.07	0.01 ± 0.005
	MLP + NS	0.08 ± 0.01	–
	MLP + mixup + NS	0.14 ± 0.006	0.07 ± 0.03
	MLP + PLANS	**0.23 ± 0.02**	0.24 ± 0.03
	MLP + PLANS + mixup	0.16 ± 0.01	**0.25 ± 0.01**
	MLP + PLANS + balancing	**0.23 ± 0.02**	0.23 ± 0.05
	MLP + PLANS + balancing + mixup	0.16 ± 0.01	**0.25** ± 0.02

The evaluation metric of the best performed model is highlighted in bold. The upper part shows the results for the CYP450 dataset and the lower part shows the results for the Tox21 dataset

### Balance the training dataset with augmented data

**Fig. 2:**
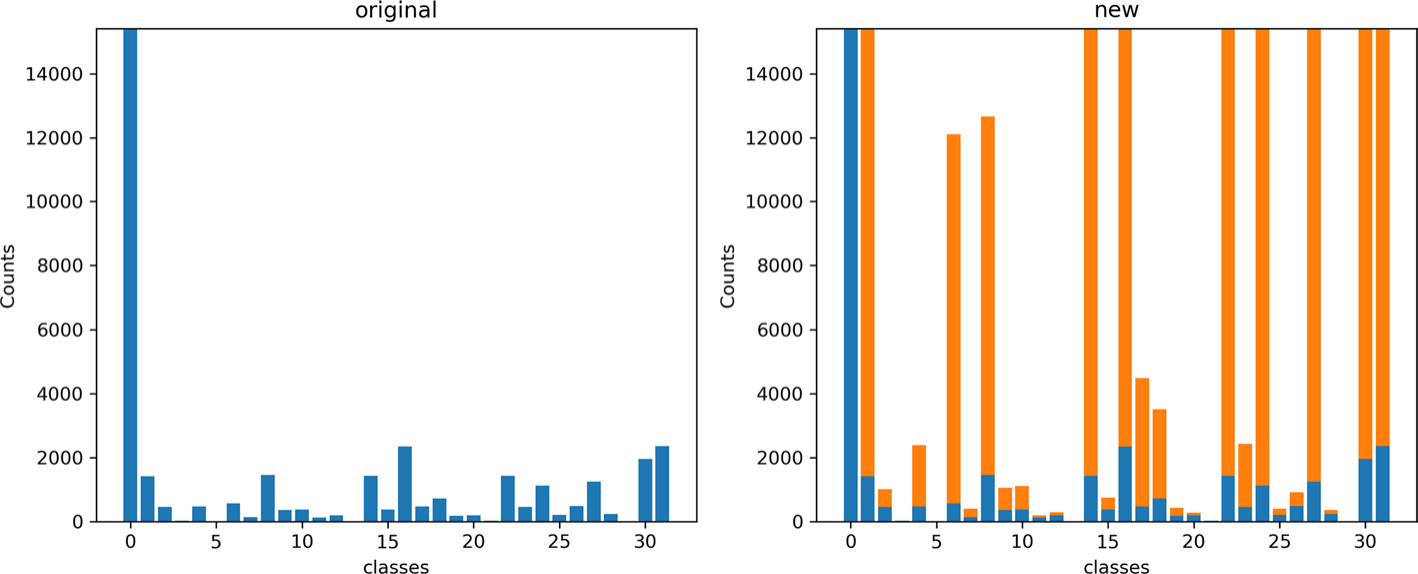
Sample distribution before and after data balancing. Blue bars represent the original samples. Orange bars represent the samples added from the ChEMBL24 dataset

Out training dataset is highly imbalanced. As shown in Fig. 2A, the fully labeled dataset has much more all-negative data than other classes. To solve this problem, we introduced an augmented labeled dataset to balance our training set using unlabeled data. The unlabeled data were labeled with a trained teacher model and added to the training set except for the samples that were labeled as all-negative. The maximum number of samples in each class was capped with the number of samples in the all-negative class. Details of data balancing can be found in the training data balancing in “Methods” section After balancing, the labels were more evenly distributed (Fig. 2B). However, the classes that had too few samples still could not be balanced to the same level as other classes. A possible solution is to introduce more noises when training the teacher models. That will be left to our future work.

The performance of the model trained with a balanced training set was shown in the last two rows of the CYP450 part and the Tox21 part of Tables 1 and 2. For the CYP450 dataset, the accuracy and recall improved 2.1% (*p* value = 0.031) and 2.6% (*p* value = 0.007), respectively, compared with the model trained with unbalanced dataset (Table 2). There are no significant changes in precision and F1. The insignificant improvement by the data balancing strategy here may be due to the ignorance of label dependency. Due to the evolutionary relationships between CYP450s, the labels generated from the one-hot encoding are not evenly distributed in nature. It is subject to future studies to take the label relationship into account. For the Tox21 dataset, the balancing method did not show a significant improvement. The reason for it could be the multi-label profile of the Tox21 dataset. A more suitable algorithm would be developed for the multi-labeled datasets in our future work.

**Fig. 3:**
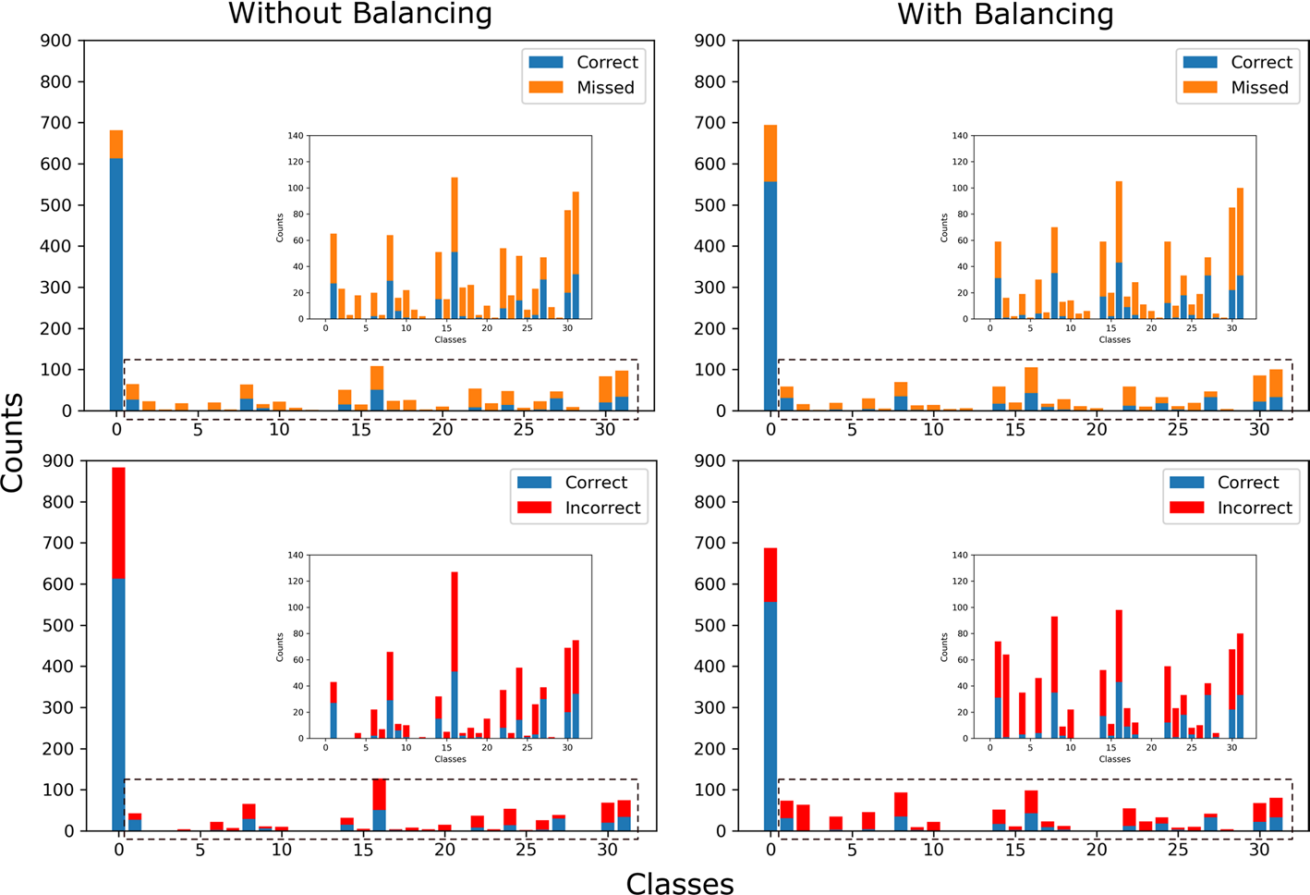
Analyzing the training results with or without the data balancing. Blue bars represent the samples that were correctly predicted. Orange bars represent the samples that the model failed to recall. Red bars represent samples that were incorrectly classified into the class by the model. The subpanels are the zoom-in of the classes without the all-negative class

In Fig. 3 we analyzed the prediction results of the model trained with or without balanced data. The upper two panels showed the model trained with balanced data missed more samples that belong to the all-negative class while missing fewer samples in other classes. In contrast, the lower two panels showed that the model made fewer mistakes in classifying samples into the all-negative class while making more mistakes in classifying samples into other classes. The summation of these two effects made the model perform better than the model trained with unbalanced raw data.

## Discussion

Lacking labeled data and an imbalanced dataset are two general difficulties in supervised learning with chemical and biological data. The difficulties are hard to overcome from the experimental side because of the immense cost of efforts and time for large-scale experiments. It makes developing robust models that can learn useful information from small and imbalanced datasets a meaningful and important direction. In this work, we developed a self-training algorithm, Partial LAbeled Noisy Student (PLANS), and applied it to an enzyme-affinity-prediction task. Our experiments proved the algorithm not only outperforms the conventional machine learning algorithms, more importantly, it also improves the performance of MLP by a large margin. Moreover, PLANS provides a way of exploiting partially labeled data as well as using unlabeled data to augment and balance labeled data.

Despite the promising results of PLANS, there is one critical problem that remains unsolved. The two most important operations of the NS method are introducing noises and utilizing abundant unlabeled data when training the student models [11]. However, other than random dropout, the most widely used data augmentation methods for images, like rotation and scaling, do not apply to chemical compounds. Thus, in our work, we used mixup to perform data augmentation, and the method worked well in most cases. Nevertheless, we noticed mixup conflicts with the usage of a large amount of unlabeled data. When we used the whole ChEMBL24 dataset without capping it with the largest class in our training set together with mixup to train the model, the performance is worse than using only the mixup. The reason could be mixing model-generated labels introduces more noise than useful information. We partially overcome it by only using a small portion of the unlabeled data to balance our training set. In the future, if a better solution other than mixup can be found to accomplish data augmentation task for chemical data, utilize a large amount of unlabeled data should be more informative and further improve the performance of PLANS trained model. The NS model represents one of the state-of-the-art semi-supervised learning methods but has not been fully explored in chemoinformatics. The proposed PLANS significantly outperformed NS in our benchmark studies and could shed light on the application of semi-supervised learning to drug discovery. It will be interesting to compare PLANS with other semi-supervised models such as FixMatch [28], Meta Pseudo Labels [29], and LaplaceNet [30] in our future work.

To make the MLP model easier to train and to incorporate substructure information into the chemical embedding, we condense the sparse ECFP by pre-training a GIN model with ECFP as labels and take the intermedium 300-dimension continuous-valued hidden layer output to use as input of the MLP model. With the smaller input, the number of parameters of the MLP model also reduced, thus, easier to converge. Moreover, the GIN model takes graph-structured chemical compounds as input, which is more informative than ECFP. It is different from autoencoders that are learned from reconstructed ECFPs or molecular graphs. In our recently published work [18], we demonstrated that the embeddings derived directly from molecule graphs would outperform conventional GNN based autoencoders. Another advantage of the fingerprint generated with GIN is that the fix sized fingerprints are suitable for the mixup, which, in our experiment, makes the training converge steadier and boosts model performance. In contrast, the mixup method is hard to use on graph structure data. The GINFP provides a novel way to describe a chemical compound with fixed length and dense, thus smaller vectors with the substructure information.

## Conclusion

To better exploit chemical molecules as input for machine learning algorithms, we developed a graph neural network-based embedding method GINFP that can convert chemical molecules to continuous-valued vectors with substructure information. In our experiments, not only deep learning methods but also conventional machine learning methods such as random forest, SVM, and XGBoost benefitted from GINFP. Furthermore, we developed a model agnostic self-training method PLANS that could be applied to any deep learning architectures to exploit partially labeled and unlabeled data. PLANS can predict the missing labels with a teacher model and iteratively improve the predictions with a series of student models. Since label sparsity is a common issue for drug-related datasets, PLANS-GINFP could serve as a general solution to improve the predictive modeling for drug discovery.

## Methods

### Overview of PLANS

**Fig. 4:**
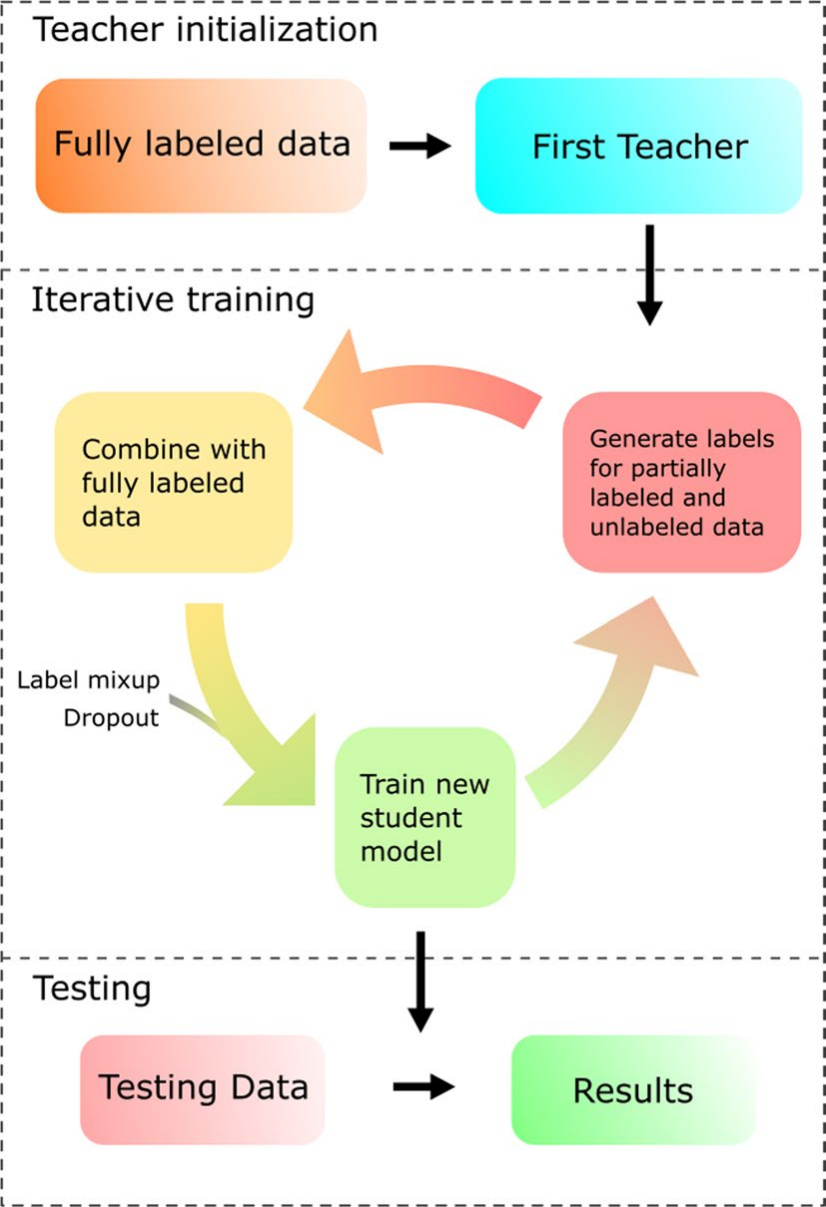
Overview of the workflow

As shown in Fig. 4, the workflow of PLANS includes teacher initialization, iterative training, and final testing. In the initialization stage, we train the first teacher model with only the fully labeled data. Then, in the iterative training stage, we use the trained teacher model to generate labels for partially labeled and unlabeled datasets, i.e., pseudo-labels. Data balancing can be introduced at this stage if needed. The fully labeled data and data with pseudo-labels are combined to train a noisy student model. Then the noisy student model is used as a new teacher model to generate labels for the partially labeled and unlabeled data. This step can be repeated until the performance of the noisy student model does not further improve. In the testing stage, we simply use the last student model to predict labels for the testing data. In the whole process, the noises are introduced by label mixup (see below) and dropout, and used only when training the student models. They are disabled when using the model as a teacher to generate pseudo-labels.

### Extended connectivity fingerprint (ECFP)

Extended connectivity fingerprint (ECFP) is a popular method for chemical structure information embedding [31]. It converts chemical structures with different lengths to a fixed-length binary vector, which reserves most bonds and atom types information. Thus, ECFP is widely used as an input feature for machine learning, especially deep learning models. To minimize the frequency of bit collision, we chose a vector length of 2048, which will result in a relatively sparse vector so that the identifiers are less likely to overlap. We used RDKit to convert the simplified molecular-input line-entry system (SMILES) strings to 2048 bits vectors with the radius parameter set to 4 [32]**.**

### GIN fingerprints (GINFP)

**Fig. 5:**
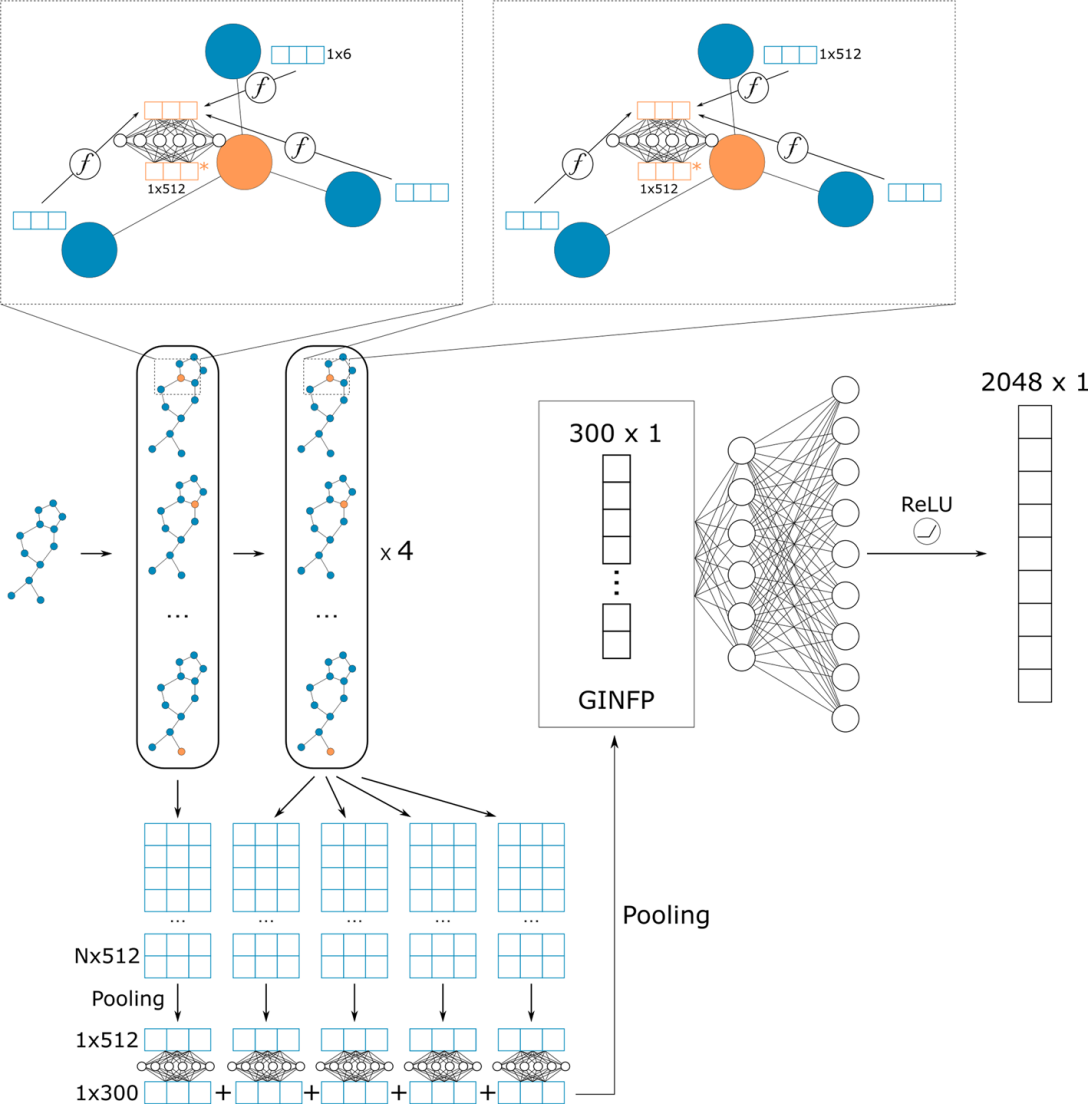
GIN model architecture and GINFP. Sum is used for node-level pooling and mean is used for graph-level pooling

GIN fingerprints are 300-dimension continuous embeddings generated by a pre-trained Graph Isomorphism Network (GIN). The architecture of the GIN layers used in our work is the same as in [15] with 1024 and 512 hidden units MLPs for node embeddings at each layer (Fig. 5). Node information update following the rule:

$$f(k, {h}_{v}, {h}_{u})=\left(1-{\epsilon }^{\left(k\right)}\right){h}_{v}^{(k-1)}+{\sum }_{u\in \mathcal{N}(v)}{h}_{u}^{(k-1)}$$

where $\epsilon$ is a trainable coefficient variable, $h$ is the node feature, $\mathcal{N}(v)$ is the set of neighbors of node $v$. It is worth noting that the properties of the neighbor node are added together in the aggregation step. That makes GIN a powerful architecture that can distinguish nodes with a different number of neighbors. It is especially important for graphs like chemical molecules which have many similar nodes. GIN is still the state-of-the-art model in chemical molecule classification tasks [9]. Node embeddings from all graph convolutional layers are added together followed by a mean pooling function to get the graph level embedding. In the pretraining phase, the graph embedding is fed into a two-layer MLP to output a 2048-dimension vector. The ChEMBL24 dataset is used to train the GIN model. The chemical compounds were converted to graphs with atom type, atom degree, atom formal charges, chirality, aromatic type, and hybridization type as node features and chemical bonds as graphs edges. ECFPs with radius 4 and length 2048 were created for the whole ChEMBL24 dataset to be used as labels in pretraining. We use binary cross-entropy loss as the criterion to calculate the gradients. The pretraining was terminated when the validation loss converged, and the pre-trained GIN model was used to generate embeddings/GINFPs for the CYP450 dataset.

### Self-training with noisy student

Knowledge distillation with self-training was used to reduce the size of models when the method was proposed [10]. Typical self-training uses a large teacher model trained with a true/sparse labeled dataset to generate soft/continuous labels for the same dataset. One or plural smaller student models are trained with the soft labeled dataset. The major reason why this model works is the continuous labels are more informative than the sparse label. They tell the student model which samples are “easier” to classify with labels that are closer to 0 or 1 and which samples are harder to classify with labels closer to 0.5. Thus, though the student models have fewer parameters than the teacher model, they can “memorize” a similar amount of information as the teacher model and reach a comparable accuracy rate. Xie et al. utilized the self-training method in an opposite way [11]. In their work, they used a small teacher model to train larger student models and make the student models perform not comparable but better than the teacher model. To make the method work, they introduced a large number of unlabeled images other than the labeled training images. They labeled these unlabeled outside images with the pre-trained teacher model and used these images to train the student models combined with the labeled images. In addition, another key method used in the work was introducing “noise” when training the student models by applying data augmentation to the inputs and dropout to the model parameters. In this report, we used a similar strategy to recurrently train teacher and noisy student models. However, unlike images, we could not use random rotation, scaling, etc. to introduce noises into the training samples, which are chemical compounds. Thus, we used partial labeling and label mixup instead.

### Neural network model

**Fig. 6:**
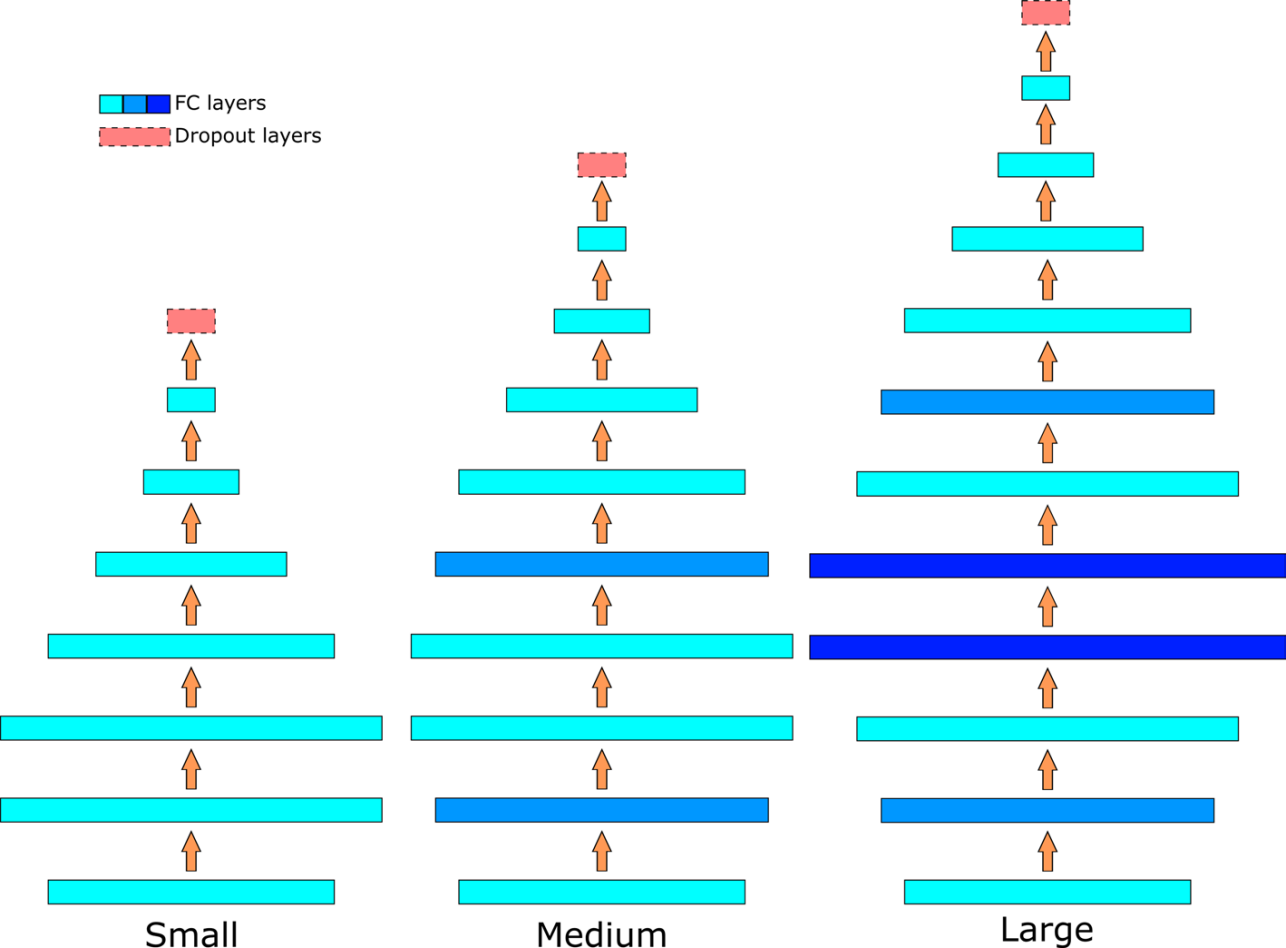
Architectures of the MLP models

To prove the feasibility of Noisy Student in the pharmacology field, we used the most basic MLPs to attenuate the influence from the model architectures. The Small, and also the initial teacher model that we used have seven hidden layers with 2x, 4x, 4x, 2x, 1x, 0.5x, and 0.25x  input length hidden units, respectively. The Medium model adds two layers with 3x input length hidden units on top of the Small model after layer 1 and layer 3. The Large model, in addition to the Medium model, inserts two layers with 6x input length hidden units after layer 2 of the Small model. A dropout layer with a drop rate equals to 0.3 is added before the final output layer in all three models during the training. The rate of the dropout was kept at 0.7 in all the experiments. All the models are implemented with Tensorflow [33]. Detailed architectures of the models can be found in Fig. 6.

### Partial labeling

To fully exploit the information in the partially labeled dataset, we use the following approach to generate pseudo-labels for the partially labeled data. First, we infer the possible class of the sample based on the partial label. For example. if the label is [0, 1, _, 0, 0] where “_” represents the missing label, the possible class of the sample will only be 8 (corresponding to label [0, 1, 0, 0, 0]) or 12 (corresponding to label [0, 1, 1, 0, 0]) of the one-hot encoding. The rest digits of the one-hot label for this sample are 0. Then we predict the 32 digits one-hot label using the teacher model. Only the value at positions 8 and 12 are taken from the predicted label and normalized, making the summation of the labels to 1. The normalized values are filled into the missing positions to generate the integral label for the partially labeled sample.

### Label mixup

“Noise” is a key factor to make the Noisy Student method work. Without noises, the classification accuracy drops around 0.7 percent in the ImageNet task [11]. However, in our case, introducing noises is not a trivial task. ECFP fingerprint [31] and GINFP (see GIN fingerprints in “Methods”section for details) that are used to represent chemical molecules are invariant to stereo transformations, so random rotation is not applicable. Chemical molecules have fixed sizes, so scaling is not applicable neither. To overcome the difficulty, we utilized an augmentation technique named mixup [34]. What it does is randomly pick two inputs, add both the inputs and the labels together with a coefficient sampled from a beta distribution. Concretely, the mixup method can be represented by the following equations:

$$\stackrel{\sim }{\mathrm{x}}=\lambda {x}_{i}+\left(1-\lambda \right){x}_{j},$$

$$\tilde{y }=\lambda {y}_{i}+\left(1-\lambda \right){y}_{j},$$

where $\tilde{x }$ and $\tilde{y }$ are mixed input and label. $\lambda$ is a coefficient sampled from beta distribution $Beta(\alpha , \alpha )$, $\alpha \in (0, +\infty )$. ${x}_{i}$ and ${x}_{j}$ are the fingerprint vectors of two randomly sampled chemical molecules. ${y}_{i}$ and ${y}_{j}$ are the labels of the molecules. We tested $\alpha =0.2$ and $\alpha =0.4$ but did not find significant performance differences. For all the experiments in this report, the value of $\alpha$ is 0.4 if not specifically indicated.

### Training strategy with partial labeled noisy student PLANS

We split the fully labeled CYP450s dataset into training and testing sets with a ratio of 7: 3. With the training set, we first train a small model as the initial teacher model. Then we use the teacher model to generate pseudo labels for the partially labeled CYP450 data and unlabeled ChEMBL24 data. Followed by that, we combine fully labeled data, partially labeled data, and outside unlabeled data to train the Medium model. The trained Medium model is used to generate the labels for the partially labeled and unlabeled samples the same as described above. These regenerated samples and the fully labeled data are finally used to train the large model. The loss function used in our training is cross-entropy as below:

$$-{\sum }_{i=0}^{N}{y}_{i}\mathrm{log}({p}_{i})$$

where $N$ is the number of classes, ${y}_{i}$ is the label of the *i*th class, ${p}_{i}$ is the predicted possibility that the sample belongs to the *i*th class. All the training is done on a single Nvidia Tesla V100 GPU. Every experiment was repeated 5 times by randomly splitting the fully labeled dataset. The training is terminated when the validating loss is stable. Partially labeled data and unlabeled data were only used for training.

### Baseline models

In addition to comparing the Large model to the Small model, we also compared the MLP model to some conventional machine learning methods, including support vector machine (SVM), random forest (RF), AdaBoost, and XGBoost. SVM, RF, and AdaBoost models were implemented with scikit-learning [35] while the XGBoost model was implemented with the xgboost package [36]. We did hyperparameter tuning with grid search for all the models. The value ranges we screened and the best values of hyperparameters for each model can be found in Table 3.

**Table 3:** Hyper parameter screening for conventional models

SVM				
Hyper parameters	C	Kernel function	$\gamma$	Degree
Screened range	[0.5, 1.0]	RBF, Sigmoid, Polynomial	Scale, auto	[2, 6]
Best	1.0	RBF	Scale	N/A

RF				
Hyper parameters	# estimators	Max depth	Max features	Min samples per leaf
Screened range	[100, 2000]	[5, 60]	[11, 2048]	[1, 20]
Best	2000	20	200	1

AdaBoost				
Hyper parameters	# estimators	Max depth	Max features	Learning rate
Screened range	[500, 2000]	[15, 35]	[512, 2048]	[0.1, 0.5]
Best	500	25	1024	0.5

XGBoost					
Hyper parameters	$\lambda$	$\gamma$	Max depth	# rounds	Learning rate ($\varepsilon$)
Screened range	[0, 3]	[0, 16]	[5, 25]	[10, 25]	[0.1, 0.5]
Best	1	1	20	21	0.2

Specifically, for SVM, we used the C-Support Vector Classification (SVC) model. Hyperparameter C is a regularization parameter. $\gamma$ is the coefficient for kernel functions. “scale” means the coefficient was decided by the number of features and variance of the input data, while, in the case of “auto”, the coefficient was only decided by the number of features. The degree is the degree of the polynomial kernel function. Since the RBF function performs the best, the degree is not applicable for the baseline model we used.

In the case of RF max depth, max features and minimum samples per leaf are parameters for decision trees. The number of estimators refers to the number of decision trees used for the forest model. For max features, scikit-learning has two default options, which are “log2”, “sqrt”, referring to log2 and the square root of the number of input features, respectively. In our case, since the input has 2048 features, log2 of 2048 is 11, the max features parameter starts from 11, and also includes 45 (square root of 2048) in our grid search.

For AdaBoost, we used binary decision trees as estimators. Maximum depth and maximum features are parameters for the decision trees. The learning rate shrinks the contribution of each estimator. Because of the low efficiency and bad performance of AdaBoost, we did not do a wide range hyperparameter search for it.

In the case of XGBoost, we follow the denotation in the original paper. $\lambda$ is a regulation parameter. $\gamma$ controls the tree pruning strength. Maximum depth and number of rounds control the maximum level of trees and the number of trees to grow. The learning rate shrinks the contribution of the newly grown tree to the previous predictions.

### Evaluation metrics

For our multi-class classification task, since the labels were highly unbalanced, to better evaluate our model, in addition to accuracy, we also introduced micro precision, recall, F1, and average precision (AP) scores, which were the metrics better fit uneven dataset. To calculate these scores, the predicted one-hot labels were converted back to 5-class multi-label labels in our CYP450 task case.

### CYP450s dataset

We chose five CYP450s described above as protein targets to create our benchmark dataset [24]. The Uniprot Knowledgebase ID of these CYP450s were P08684 (CYP450 3A4), P05177 (CYP450 1A2), P10635 (CYP450 2D6), P33261 (CYP450 2C19) and P11712 (CYP450 2C9). The chemical molecules from the dataset were labeled with active or inactive based on their binding affinity to each CYP450 target. However, not all of the molecules in the dataset had an explicit binding affinity label to all five CYP450s. Some labels were missing because the experiments were not conducted. In this report, we denoted the chemical molecules in the CYP450s dataset with at least one missing label as partially labeled data and the rest chemical molecules as fully labeled data. The CYP450s dataset has 17,121 chemical molecules, among which 5162 molecules are fully labeled while 11,959 molecules were partially labeled. 11,647 compounds bind to at least one of CYP450s, and 4110 compounds specifically bind to only one CYP450. The full labels were converted to 32-class labels in which each class represents a unique combination of the five affinity labels. For instance, [0, 0, 0, 0, 0], labels of a chemical that was inactive against all five CYP450s, was converted to class 0. In another example, [0, 1, 1, 0, 1] was converted to class 13. To exploit the partially labeled data, we took advantage of the self-training strategy and generated partially confident labels, which will be described later. In the results section, we will show the partially labeled data improve the performance of the trained model with a big margin comparing to the model trained only with the fully labeled and the unlabeled data.

### Tox21 dataset

The Tox21 dataset from MoleculeNet is another baseline to test our model [37]. It contains the activities of 7,831 compounds against 12 biological targets or pathways, which are nuclear receptor(NR)-androgen receptor (AR)-ligand-binding domain (LBD), NR-AR, NR-aryl hydrocarbon receptor (AhR), NR-Aromatase, NR-estrogen receptor (ER)-LBD, NR-peroxisome proliferator-activated receptor (PPAR)-gamma, SR-antioxidant response element (ARE), stress response (SR)-ATPase Family AAA Domain Containing 5 (ATAD5), SR-heat shock factor response element (HSE), SR-mitochondrial membrane potential (MMP), and SR-p53 [38]. Similar to the CYP450 dataset, Tox21 includes many missing labels. We formulate the chemical toxicity prediction using the Tox21 dataset as a multi-label classification problem. The label for the target is positive if the chemical compound has toxicity by interacting with the target. The multi-label means one chemical compound can have more than one targets.

### ChEMBL24 dataset

The ChEMBL “is a manually curated database of bioactive molecules with drug-like properties” [39]. In our work, as the outside dataset for the Noisy Student method, we used the ChEMBL24, which was released in 2018 and has over 1.7 million drug-like chemical molecules. We used the SMILES of the chemical in the ChEMBL24 dataset to generate fingerprints. Chemical molecules that are not loadable by RDKit are dropped from the dataset. The final dataset has 1,737,165 drug-like chemical molecules.

### Construction of input graphs

**Fig. 7:**
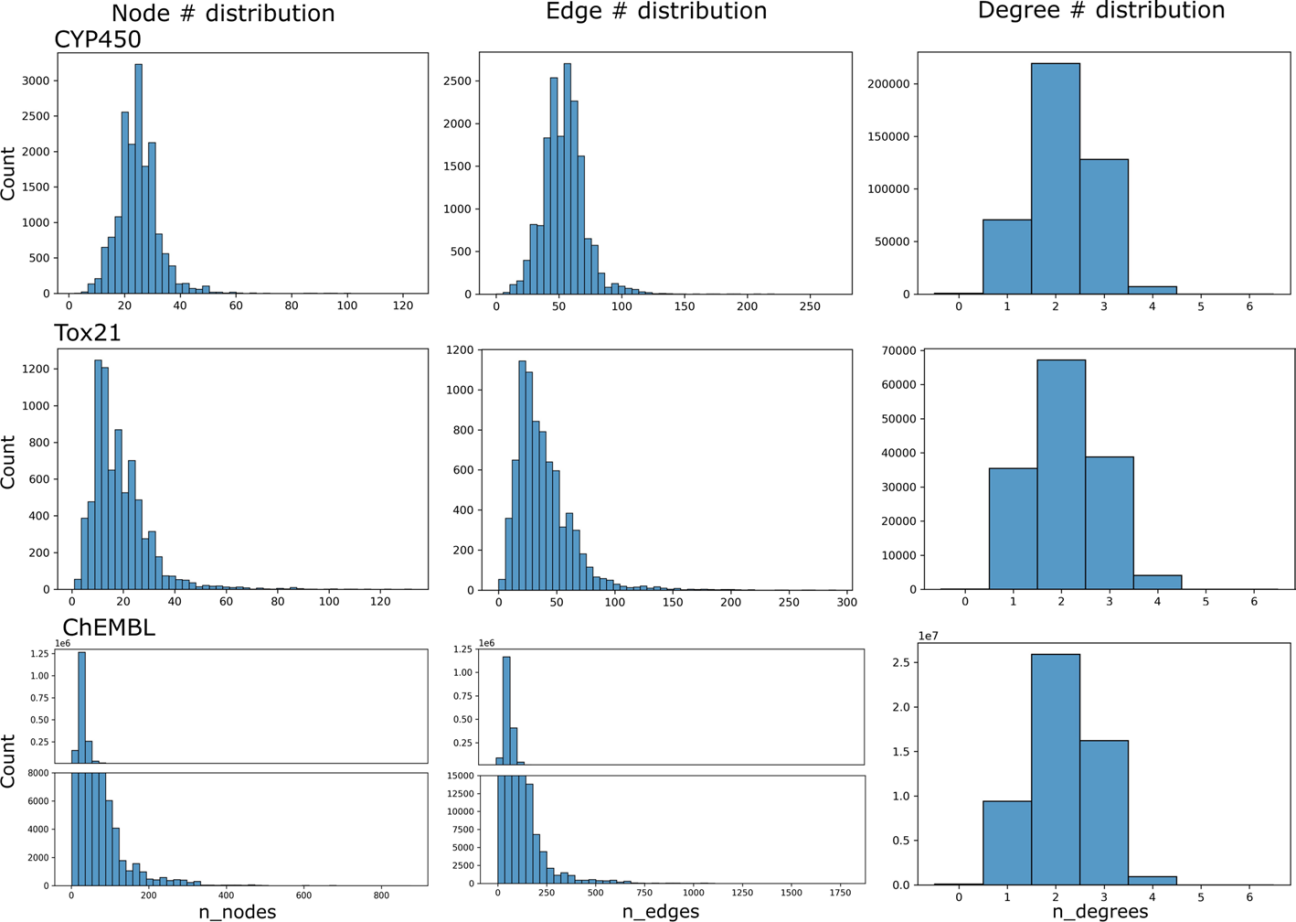
Statistics of chemical molecule graphs for datasets used in our experiments. The upper and lower parts of the first two panels for the ChEMBL dataset have different y-axis scales because of the large number of nodes/edges distribute in several bins

The graphs used to generate GINFPs were constructed from chemical molecules with atoms as nodes and chemical bonds as edges. Each node contains the chemical property of the atom. Specifically, atom type, degree, formal charge, hybridization type, aromatic, and chirality were used as node features. The number of nodes, edges, and degrees of the nodes of each dataset used in our experiments are plotted in Fig. 7.

### Training data balancing

The labels in the CYP450s dataset were unbalanced. The number of chemical molecules that did not bind to any of the five CYP450s is much larger than the number of chemical molecules that bound to at least one CYP450. In our experiment, we also tried to solve this problem by taking the advantage of self-training method. When introducing the data balancing in the experiment, the unlabeled data were not simply combined with fully labeled and partially labeled data. Instead, the procedure of adding unlabeled data to the training set was closely monitored. The sample was added to the training set only when the predicted pseudo label was not all negative and the class had fewer samples than the all-negative class. We remove all the unlabeled samples from the training set and repeated this balancing whenever a new teacher model was trained. In this study, the unlabeled data were from the ChEMBL dataset.

## Data Availability

The dataset used in this work are from a public database from https://www.ebi.ac.uk/chembl/. The processed data will be released after the manuscript get accepted. The code used to perform the experiments is available at: https://github.com/XieResearchGroup/PLANS.

## Abbreviations

PLANS: Partially labeled noisy student
GINFP: Graph-isomorphism-network-based fingerprint
ECFP: Extended-connectivity fingerprint
CYP450: Cytochrome P450
SVM: Support vector machine
RF: Random forest
MLP: Multi-layer perceptron
GNN: Graph neural network
SMILES: Simplified molecular-input line-entry system
NLP: Natural language processing
